# Antischistosomal Activity of *Origanum majorana*, *Ziziphus spina-christi*, and *Salvia fruticosa* Plant Extracts on Hamster Infected with *Schistosoma haematobium*

**DOI:** 10.1155/2021/5545331

**Published:** 2021-06-13

**Authors:** Yousef Abdal Jalil Fadladdin

**Affiliations:** Department of Biological Sciences, Faculty of Sciences, King Abdulaziz University, Jeddah, Saudi Arabia

## Abstract

World Health Organization (WHO) has approved only one treatment for schistosomiasis, praziquantel (PZQ), but some poor efficacy was noticed in patients during the early stage of infection. Therefore, researchers have intensified their efforts to research new alternative medicines to treat schistosomiasis. In the present study, *in vitro* as well as *in vivo* studies have been accomplished to evaluate the effect of *Origanum majorana*, *Ziziphus spina-christi*, and *Salvia fruticosa* extracts in a different concentration 500, 250, 125, 62.5, and 31.25 *μ*g/ml on golden hamster infected by Egyptian strains of schistosome (*Schistosoma haematobium*). *In vitro*, the adult worms and schistosomula of *S. haematobium* were investigated in RPMI-1640 medium for 48 hrs. The results showed that the concentration 500, 250, and 125 *μ*g/ml of *Origanum majorana*, and *Ziziphus spina-christi* caused dead of 100% of Egyptian *Schistosoma* strains of adult worm and schistosomula of *S. haematobium* within 6 to 12 hrs of incubation. On the other hand, the extract of *Salvia fruticosa* at concentrations 500, 250, and 125 *μ*g/ml showed death 100% parasites after 12 to 24 hrs of incubation. Inclusion, *Origanum majorana*, and *Ziziphus spina-christi* showed effectiveness against Egyptian *Schistosoma* strains (*S. haematobium*), a slight decrease in *Salvia fruticosa* was observed. Therefore, these medical plant extracts may be used as a safe and effective treatment for schistosomiasis.

## 1. Introduction

Schistosomiasis is a worldwide disease, and it is sometimes called bilharzia. The intermediate host is the freshwater snail that transmits intravascular weakening disease resulting from infection with one of the types of *Schistosoma* that lives within the human vascular system, which lives within the circulation system of people [1, 2]. The World Health Organization Special Program for Research and Preparing in Tropical Diseases has classified schistosomiasis into the second category after malaria in significance as a targeted tropical disease. The World Health Organization (WHO) has considered schistosomiasis to be a tropical disease that has been neglected. All over the world, in known transmission ranges, about 732 million people have been assessed as being defenseless to the disease [3].

There are two major shapes of schistosomiasis: the intestinal (caused by four species called *Schistosoma mansoni* (*S. mansoni*, *S. japonicum*, *S. mekongi*, and *S. intercalatum*) and the urogenital caused by (*S. haematobium*) [4].

*S. haematobium* was a chronic disease in old Egypt. Ruffer in 1910 was the first to diagnosis *S. haematobium* contaminations in mummies. He recouped calcified schistosome eggs from two Egyptian mummies of the 20^th^ Dynasty [5]. The radiological examination too unequivocally recommended that the calcified bladder in two other mummies were due to *S. haematobium* contamination [6, 7]. Also, in 1937, the primary schistosomiasis epidemiological studies were conducted over Egypt. *S. haematobium* was found as it were in Upper Egypt, which compares geographically to the southern portion of Egypt. Cases were restricted to districts with a perpetual water system, agricultural systems in the area [8]. Then, Barakat [9] found that in zones utilizing the perpetual water systems, both within the Nile Delta and Vile Valley south of Cairo, an average of 60% predominance rate of *S. haematobium* infection was evaluated. In contrast, those areas that utilized basin water systems had an assessed 6% predominance. The allocation of *S. haematobium* hence appeared a clear relationship to the improvement of irrigation plan that permitted for year circular standing water, increased human-water contact, and get better snail habitat. The predominance of *S. mansoni* was evaluated at 60% prevalence within the Northern and Eastern parts of the Nile Delta and only 6% predominance within the Nile Valley locale overviewed [9].

Side effects of schistosomiasis were caused by the body response to the eggs of schistosome. Haematuria (blood in urine) appears as a classical indicator of urogenital schistosomiasis. Fibrosis of the ureter and bladder and kidney harm is in some cases diagnosed in progressed cases. Cancer of the bladder is another conceived complexity within the last stages. In ladies, urogenital schistosomiasis might current with genital injuries, nodules in the vulva, suffering in sexual intercourse, and vaginal bleeding. In man, schistosomiasis may also be associated with the urogenital system, diseases of the prostate, seminal vesicles, and other organs. In the long term, this disease may also have other irreversible outcomes, including infertility [4].

Until the 1970s, the treatment of schistosomiasis was complicated and nearly poisonous until praziquantel was discovered [10, 11]. In 1988, the World Health Organization approved the praziquantel as a treatment and control of schistosomiasis [11]. Though praziquantel is considered safe and successful against all *Schistosoma* species [12] and has been used in the last 32 years, it is considered a long period for a single drug for the parasite; the drug is not free of troubles. For occurrence, the enormous and elite utilize of praziquantel for numerous decades as a sole medicate has clearly raised legal fears that schistosomes maybe become resistant to praziquantel sooner or afterward show up [12]. Moreover, praziquantel works successfully against adult worms of schistosomes, but it is inactive against other stages of schistosomes, such as schistosomula, preadults, and juvenile adults. As a result, the recurrence of the drug for some time is essential to murder those parasites that have since developed. In addition, having a single medicate to treat an illness that influences millions of individuals in numerous geographical ranges may be a genuine concern. Subsequently, it is urgent to improve unused successful and secure antischistosomal drugs. The advancement of antischistosomal mechanisms from natural sources has become a critical requirement in recent years. Medicinal plants have been a natural source of medicines for thousands of years [13]. The medicinal plant has been utilized as natural sources for the discovery of unused medicates. The scientific assessment of therapeutic plants utilized within the planning of people treatment has given present-day pharmaceutical with successful pharmaceuticals for the treatment of infections caused by parasites. Several natural extracts or compounds have been distinguished from plants with promising antischistosomal properties [14–21]. In this setting, the medicinal plants and compounds derived from these plants acquire an unmistakable quality as conceivable sources of modern drugs within the treatment and control of schistosomiasis.

The present research aimed to evaluate the aqueous extracts of *Origanum majorana*, *Ziziphus spina-christi*, and *Salvia fruticosa* against Egyptian strain of schistosome (*Schistosoma haematobium*) in the laboratory as well as in the experimental animals.

## 2. Materials and Methods

### 2.1. Plant Materials

The leaves of *Origanum majorana*, *Ziziphus spina-christi*, and *Salvia fruticosa* were obtained, which were washed well to prevent the loss of active ingredients. They were dried in the shade and avoided exposure to sunlight as they were identified by the Department of Horticulture, Faculty of Agriculture, Ain Shams University.

### 2.2. Aqueous Extract Preparation

The extraction of *Origanum majorana*, *Ziziphus spina-christi*, and *Salvia fruticosa* leaves was prepared according to Ekpo and Etim [22]; by an electric grinder, the dried plant leaves were ground to fine powders. In two liters of distilled water, the ground leaves weighing 200 (g) were extracted (1 : 10 *w*/*v*) by cold extraction and evaporation of the extract *in vacuo*. Using a rotary evaporator at 40°C, the extract was concentrated *in vacuo*. Finally, the extract was placed in porcelain dishes in a temperature-controlled oven to remove the remaining water from the extract and to get a residue. The residue of the three plant extracts was approximately 8.5 g for each extract. The extracts at 4°C were stored for use later [22].

### 2.3. Toxicological Study

To measure the maximum nontoxic concentration (MNTC), Serial dilutions of 10-300 *μ*g/ml of the plant extracts onto Vero cells according to Mosmann technique [23] were applied. When the aqueous extract is diluted in a manner that preserves the normal shape of the tested Vero cells as well as their density, by comparing them with untreated control cells, at least 95% of the optical density is the MNTC value.

### 2.4. Experimental Design

#### 2.4.1. Infection of the Hamster with *Schistosoma* Cercariae

Hamsters were ready at the start of the biological experiment (Week 0), thus losing consciousness and suppressing muscle relaxation and reflex activity with general anesthesia for the hamster. To provide a combined effect for anesthesia, a volume-based ratio 3 : 1 ketamine and Rompun was used. Intraperitoneally, the dose of anesthesia was injected 0.02 ml/30 g of the animal's body weight. The hamster was arranged on a wooden shelf after the animal was shaved from the stomach area. To allow easy penetration of the infected stage (cercariae), cotton wool was dipped in water and was used to moisten the shaven area. On each hamster, a metal ring of 1 cm was placed on the shaven area of hamster; then, using a micropipette, a suspension containing around 250 live infective stage (cercariae) was placed in the metal ring for 30 minutes to give allowance to the cercariae it to penetrate the hamster [24].

### 2.5. Parasite

Pairs of Egyptian strains of schistosomes adult worms (*S. haematobium*) were obtained from sacrificed infected hamster by perfusion of the mesenteric veins and hepatic portal system of their livers using citrated saline after three months of postinfection according to Stirewalt and Dorsey technique [25].

### 2.6. *In Vitro* Study

Perfusion worms were washed three times with the RPMI 1640 (BioWhittaker®) culture medium (Lonza, B-4800 Verviers, Belgium), which was used for culturing the parasite. 20% fetal calf serum, antibiotics, and L-glutamine (300 IU penicillin, 160 *μ*g gentamycin per ml and, 300 *μ*g streptomycin) were used as supplements for the medium [26]. After the washing, in each well of a 24-well culture plate, 7 couples of worms were transferred (TPP, St. Louis, MO) having one (ml) of the RPMI 1640 medium. One (ml) of the concentrations (500, 250, 125, 62.5, and 31.25 *μ*g/ml) from each examined aqueous plant extract were added. The final volume was 2 (ml) in each well. The plate in a humid atmosphere containing 5% CO_2_ at 37°C was incubated (Thermo Fisher Scientific, Marietta, OH, USA) [27]. The parasite was kept for 48 h. 10 *μ*g/ml of PZQ used as a positive control, while a medium with pure medium and sterile distilled water were used as negative controls. Under a sterilized laminar flow chamber, all previous steps were performed. The trial was carried out in repeated and triplicate 3 times. An inverted optical microscope (Olympus CK2) was used to monitor the treated worms' mating (pairing), motility rate (activity changes of worm's motor), and mortality. Worms that did not show motility for two minutes were considered dead. Changes in worm's motor activity (motility) of schistosomes were assessed qualitatively and decreased motor activity was defined as “significant” or “slight” [28]. The adult schistosomes and their schistosomula observation was performed, throughout the 48 h, experimental incubation period, and the results were recorded at 2, 4, 6, 12, 24, and 48 h (trial endpoint for the negative control groups).

### 2.7. Electron Microscope

To observe the morphological changes that occur in the tegument of adult worms in both male and female after being exposed to plant extracts at different concentrations, they were prepared for examination by Scanning Electron Microscope (SEM) according to Glauert technique [29].

### 2.8. *In Vivo* Study

#### 2.8.1. Animals

A total of twenty-six adult golden hamsters weighing 105–130 g infected with *Schistosoma haematobium* were maintained and kept separately in a cage prepared for the examination of fecal materials for obtaining parasites in the study. The infected animals were randomly divided into 6 groups with 5 animals each; at the time of the experiment, the route of administration was orally for all treatments:

(G1) Uninfected golden hamster (Healthy control).

(G2) Infected golden hamster with adult *S. haematobium* and schistosomula given distill water (negative control).

(G3) Infected golden hamster with adult *S. haematobium* and schistosomula treated with 200 mg/kg PZQ (positive control).

(G4) Infected golden hamster with adult *S. haematobium* and schistosomula treated with 600 mg/kg *Origanum majorana*.

(G5) Infected golden hamster with adult *S. haematobium* and schistosomula treated with 600 mg/kg *Ziziphus spina-christi*.

(G6) Infected golden hamster with adult *S. haematobium* and schistosomula treated with 800 mg/kg *Salvia fruticosa*.

### 2.9. Histopathological Assessment

After dosing, the hamsters were slaughtered to obtain the liver, spleen, and kidney and were stored in 10% formalin for at least two weeks. Small portions of the organs were obtained and washed overnight under running water to remove excess formalin. Slices were made to be examined microscopically for granulomas according to Baker [30] and Farah [31].

### 2.10. Identification of Most Potent Plant Extract

Identification of antischistosomal activity of three plants: *Origanum majorana*, *Ziziphus spina-christi*, and *Salvia fruticosa* evaluated at specialized Regional Center for Mycology and Biotechnology (RCMB) by extraction method, and absorbance of oil solutions in methanol measured with UV-240 spectrophotometer (Schimmadzu-Corporation, Kyoto, Japan). By the maceration method, the plant extract was prepared. The appropriate solvent has been selected to make the maceration with stirring or several times shaking at room temperature [32].

### 2.11. Gas Chromatography-Mass Spectrometry (GC-MS) Analysis

Extract components were identified using a Trace GC1310-ISQ mass spectrometer (Thermo Scientific, Austin, TX, USA) with a TG-5MS direct capillary column (30m × 0.25mm × 0.25 *μ*m thickness film). The temperature of the oven column started at 50°C and was raised by 5°C/min until it reached 230°C and held for 2 min. It was raised by 30°C/min until reached 290°C and held for 2 min, which is the final temperature. The injector was kept at 250°C and MS transfer line temperature at 260°C. At a constant flow rate of 1 ml/min, use helium gas as the carrier gas. The solvent delay was 3 min, and 1 *μ*l of diluted samples were injected automatically by AS1300 Autosampler connected to the GC. In full scan mode, EI mass spectra was aggregated at 70 eV ionization voltage over the range of *m*/*z* 40-1000. The ion source temperature was fixed at 200°C. The active component was determined by comparing the mass spectra and retention time with those of NIST 11 and WILEY 09 mass spectral database.

### 2.12. Statistical Analysis

To analyze the collected data, the Statistical program (SPSS) for windows version 25.0 was used. Normality tests have been used to determine whether a particular set of data was normally distributed. The Shapiro Wilk test was used to check the normal distribution [33]. Moreover, all data were presented quantitatively. Groups were compared using one-way ANOVA to compare of quantitative data of more than two groups for parametric data. The *p* value < 0.05 was considered statistically significant.

## 3. Results

### 3.1. Cytotoxicity

When treating Vero cells with a maximum dose of nontoxicity (MNTD) for the tested plant extracts (*Origanum majorana*, *Ziziphus spina-christi*, and, *Salvia fruticosa*) of 250, 300, and 350 *μ*l/ml, respectively, no morphological differences were shown compared to the control group.

### 3.2. Phytochemistry

**Figure 1:**
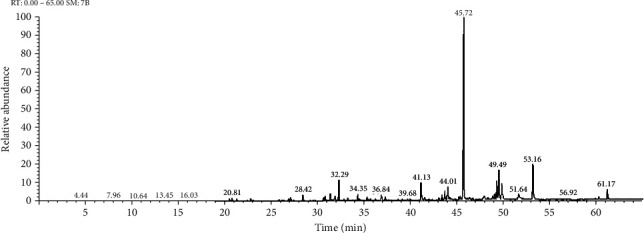
GC-MS chromatogram of aqueous extract of *Origanum majorana.*

Sweet marjoram (*Origanum majorana*) is characterized by a pleasant odor and flavor and a strong spicy. The chemical compositions of the important oils acquired from *O. majorana* (Figure 1) are listed in Table 1.

**Figure 2:**
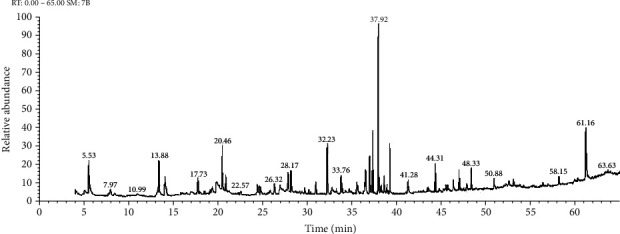
GC-MS chromatogram of aqueous plant extract of *Ziziphus spina-christi.*

On the other hand, the chemical compositions of the important oils acquired from *Ziziphus spina-christi* (L.) (Figure 2) are recorded in Table 2.

**Figure 3:**
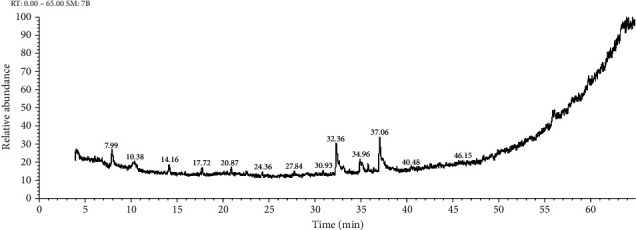
GC-MS chromatogram of aqueous plant extract of *Salvia fruticosa.*

The chemical compositions of the important oils acquired from *Salvia fruticosa* are recorded in Table 3 (Figure 3).

### 3.3. Antischistosomal Activity

In laboratory treatment effectiveness of *Origanum majorana*, *Ziziphus spina-christi*, and *Salvia fruticosa* extracts on adults of *S. haematobium* at diverse concentrations as studied, natural mating was affected by all the experimental extracts, these causing disconnections of couple *Schistosoma* relying on the concentration utilized and exposure period. About 90% of the parasites has been disconnected in the first two hours with the utilize of 500, 250 *μ*g/ml, ~80% of parasites had been detached within 4 h with the dose of 125, 62.5 *μ*g/ml, and in a lower concentration of 31.25 *μ*g/ml, the detached was 65% after 12 h of *Origanum majorana* and *Ziziphus spina-christi*. While the *Salvia fruticosa* showed less effect than *Origanum majorana* and *Ziziphus spina-christi* which the detached showed after 6 h (65%) at the highest concentration. 10 *μ*g/ml Praziquantel after the first 2 h of incubation caused separation in a pair of worms. Whereas, negative control groups were observed to separate couples after 12 hours of incubation. In contrast, concentrations that were not lethal to the parasites at 100% were an effective inhibitor of male-female couples, so upon completion of the incubation, there were no couples between the worms.

About the movement, at most concentrations, a noteworthy decrease was observed within the worm motility. The concentration of the extracts and the incubation time were directly related to the movement of the parasite as they were the main factor in decreasing the percentage of movement among the *Schistosoma* worms, after two hours of incubation; the movement activity of all adult worms was monitored, after exposure to concentrations of 500, 250, and 125 *μ*g/ml of both *Origanum majorana* and *Ziziphus spina-christi* begins to decline slightly. Whereas, the movement of adult worms began to decline after 12 hours without apparent loss of movement when exposed to concentrations of 62.5 and 31.25 *μ*g/ml. While the *Salvia fruticosa* showed less effect than *Origanum majorana* and *Ziziphus spina-christi* which reduced the movement appearance after 12 h at high concentration. Movement activity was watched at a 24 h period with no change, in negative control groups. Whereas it reduced at a 48-h period but not total lack of motility happened. Furthermore, after the first 2 h of incubation of the *Schistosoma* with 10 *μ*g/ml of PZQ led to a reduction in motility activity, and the total lack of activity occurred in all worms at a 4 h period.

**Figure 4:**
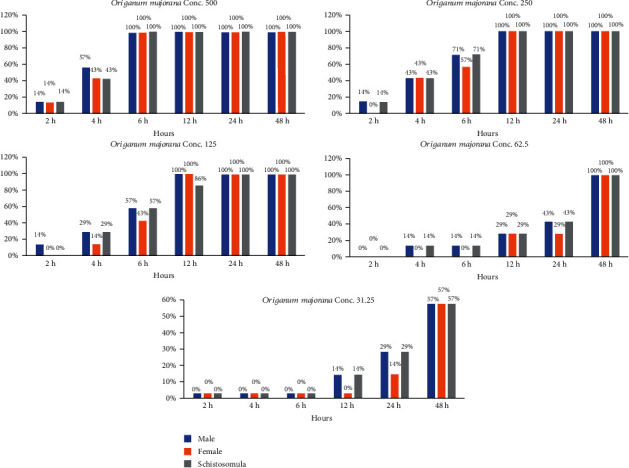
Statistical outcome of the effectiveness of *Origanum majorana* with different times and different concentrations on *S. haematobium.*

**Figure 5:**
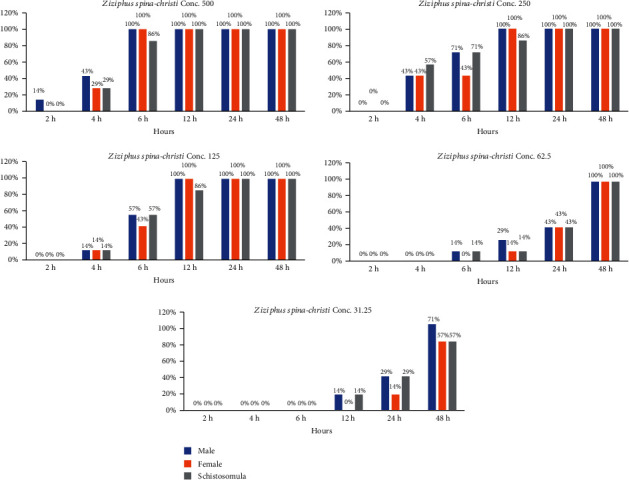
Statistical outcome of the effectiveness of *Ziziphus spina-christi* with different times and different concentrations on *S. haematobium*.

**Figure 6:**
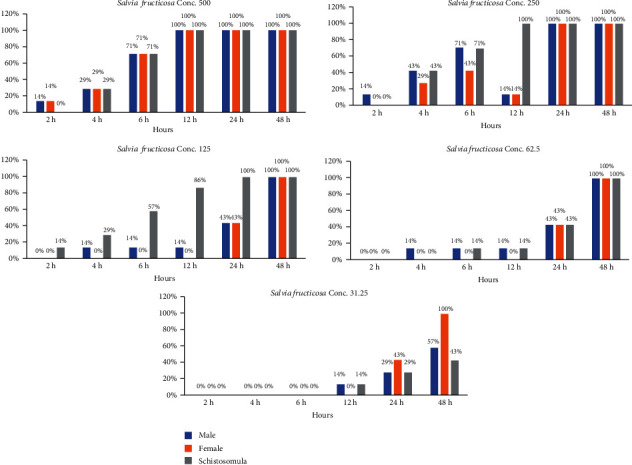
Statistical outcome of the effectiveness of *Salvia fruticosa* with different times and different concentrations on *S. haematobium*.

The current result showed that the existence of *S. haematobium* adult parasites and schistosomula exposed to aqueous extracts of *Origanum majorana*, *Ziziphus spina-christi*, and *Salvia fruticosa* depended immediately on both the incubation time and concentrations. The dose of 500, 250, and 125 *μ*g/ml of *Origanum majorana* and *Ziziphus spina-christi* caused the death of 100% of schistosomule and adult worms within six and twelve hours of incubation, respectively (Figures 4 and 5). Though, the concentrations of 500, 250, and 125 *μ*g/ml of aqueous extract of *Salvia fruticosa* resulted dead of 100% parasites after twelve to twenty-four hours of incubation, respectively (Figure 6). *Origanum majorana* and *Ziziphus spina-christi* (500 *μ*g/ml) lead to critical kill rate (*p* < 0.001) between *Schistosoma* parasites after six hours of incubation, while at 250 and 125 *μ*g/ml concentration of the *Origanum majorana* and *Ziziphus spina-christi* extracts expressed their kill-rate effect on adults *S*. *haematobium* after eight and twelve hours of incubation, respectively (Figures 4 and 5). The current result showed the adult male and female worms had a reaction with the different concentrations of the aqueous extracts used in the mortality rate or existence rates, as the results showed.

Whereas, the effect of PQZ on positive groups was 100% complete death of worms after four hours of incubation. In contrast, untreated worms lived up to 48 hours of incubation and they were the negative groups; the experiment ended at this stage.

### 3.4. Scanning Electron Microscope (SEM) Examination

**Figure 7:**
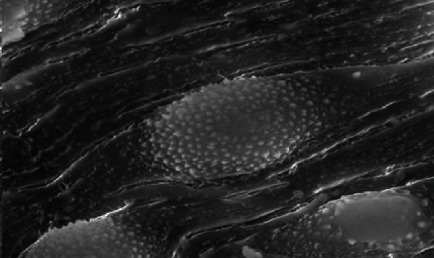
Normal tegument of *S. haematobium* from the hamster tissue.

The body wall of adult worms for *Schistosoma haematobium* was studied by scanning electron microscopy, obtained from untreated golden hamsters; in the male, the ventral canal called the gynaecophoric and the dorsal surface of the worms were characterized by the presence of many large spiny tubercles, but the areas between these tubercles have no spines; on the other hand, the abdominal surface was supported by lines of small spines. The ventral sucker appeared in a round shape and was covered with spines, while the oral sucker was oval in shape and strengthened with different sizes of sharp spines. Tubercles were surrounded by wrinkled tegument (Figure 7).

**Figure 8:**
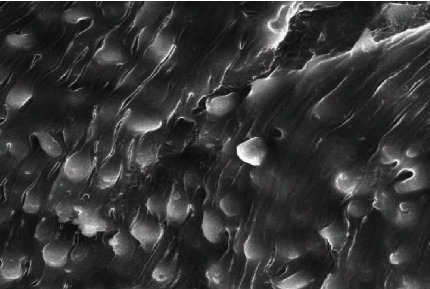
Effect PZQ on adult worms of *Schistosoma.*

After treating the golden hamster with aqueous extracts of *Origanum majorana*, *Ziziphus spina*-*christi*, and *Salvia fruticosa*, significant tegumental changes appeared in the adult worms of *S. haematobium* and schistosomule. Similarly, after incubating male and female *Schistosoma* for 48 hours in the laboratory at concentrations of 500%, 250%, 125%, 62.5%, and 31.25% of plant extracts, ultramorphological differences were observed in adult worms of males and females, with more harmful effects on males. When comparing the worms exposed to *Origanum majorana*, *Ziziphus spina*-*christi* with the untreated group revealed a varying degree of morphological changes. Whereas, the worms that were treated with PQZ revealed a change in the tegument of the worms by 100%, which showed a similarity between the effect of the extracts and the positive control (Figure 8).

**Figure 9:**
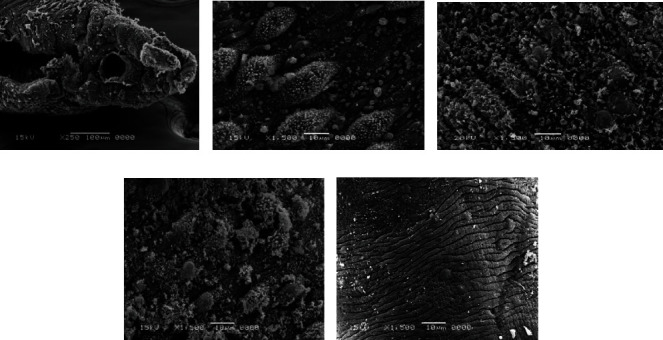
Scanning electron microscopy of *S. haematobium* (adult worms and schistosomula) after exposure to different concentrations of *Origanum majorana*. (a) Destroyed sucker. (b, c, d) Dorsal surface of male showing tegumental exfoliation with damage and exfoliation of spines and tubercles. (e) Schistosomula.

**Figure 10:**
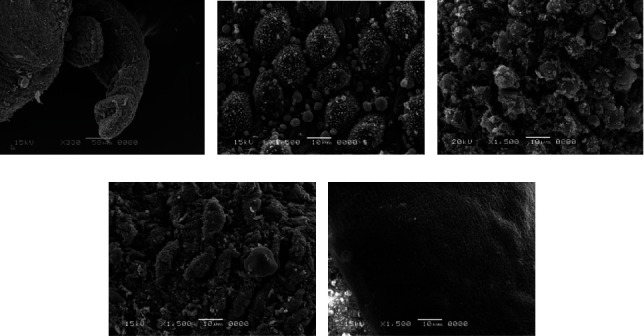
Scanning electron microscopy of *S. haematobium* (adult worms and schistosomula) after exposure to different concentrations of *Ziziphus spina-christi*. (a) Destroyed sucker. (b, c, d) Dorsal surface of male showing tegumental exfoliation with damage and exfoliation of spines and tubercles. (e) Schistosomula.

Male *Schistosoma* had shown clear morphological changes, especially on the dorsal surface, with the appearance of deformations in the tubercles and the decay of the spines (tegument sloughing or peeling, peeling of tubercles, spines, devastation). Changing or destroying the suckers was observed, where the oral sucking of some worms was deformed, and small bubbles with variable numbers appeared around the morphologically modified tubercles. Whereas in females, the effect was less due to the absence of tubercles or spines, so wrinkles, erosion, and peeling (contraction and peeling of the dorsal surface) were observed, as well as the destruction of suckers or a change in their shape (Figures 9 and 10).

**Figure 11:**
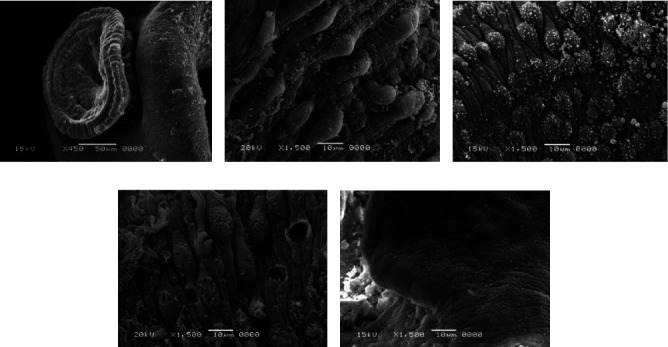
Scanning electron microscopy of *S. haematobium* (adult worms and schistosomula) after exposure to different concentrations of *Salvia fruticosa*. (a) Destroyed sucker. (b, c, d) Dorsal surface of male showing tegumental exfoliation with damage and exfoliation of spines and tubercles. (e) Schistosomula.

In connection with *Salvia fruticosa* extract, the worms (adult worms male and female, schistosomule) emerged comparable morphological tegumental changes but with a lower degree to *Origanum majorana* and *Ziziphus spina-christi* stimulate morphological changes (Figure 11).

### 3.5. Histopathological Evaluation

**Figure 12:**
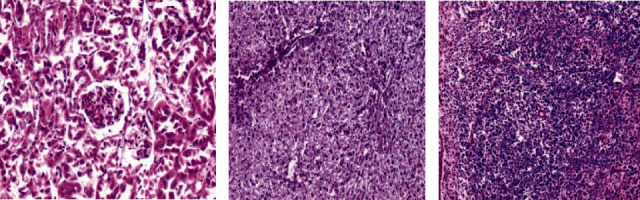
Histological section staining with hematoxylin and eosin for a healthy control (negative control). (a) Normal cortical structure in kidney control (×200). (b) Normal hepatic lobular architecture in liver control (×100). (c) Normal architecture in spleen control (×100).

Histological examinations by light microscopy were performed for the healthy group (negative control) of the liver, kidney, and spleen section. In the kidney, several renal corpuscles were present in the cortical parenchyma of the kidney as well as it contained the proximal and distal renal tubule (X200) (Figure 12). While the portal tracts appear extending from the central vein to the periphery of the hepatic lobules (X100) (Figure 8). In addition, the spleen appears in a normal structure, consisting of a thickened capsule of connective tissue which consisting of the red pulp and the white pulp. In the central region, a wealthy area with T cells, also B cell important for follicles surrounded by a periarterial lymphoid sheath, the white pulp was collected around the artery surrounded by basic follicles rich in B cells. In the area around the red and white pulp, they were isolated by peripheral sinuses implanted in a layer of lymphocytes (X100) (Figure 12).

**Figure 13:**
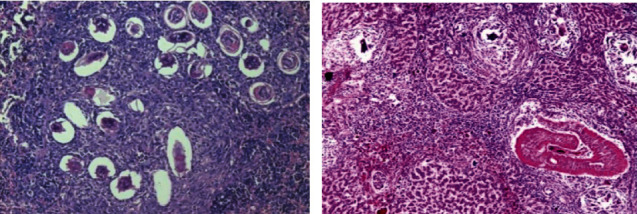
Histological section staining with hematoxylin and eosin for infected untreated control. (a) Aggregate of deposited ova of *Schistosoma* in spleen, enclosed by lympho-epithelioid tissue reaction (×200). (b) A worm affect inside a portal vein and multiple egg granulomas in liver (×100).

Moreover, the parasite did not demonstrate any clear effect on the kidney in the infected untreated control group, and all its tissues were appeared normal; nevertheless, the effect of the parasite appeared on both the spleen and liver, which appeared in hepatic parenchyma due to chronic granulomatous injuries. Injuries were formed by several *Schistosoma haematobium* eggs that contain mature miracidia, which contain many epithelioid cells, plasma cells, eosinophils, macrophages, and chronic inflammatory cells in the form of lymphocytes that formed a granuloma with a severe area of fibrosis (Figure 13). In the spleen, the boundaries between the red and white pulp began to disappear due to the cause of the appearance of the eggs surrounded by the inflammatory cellular response. A small number of spleen cells were evaluated. The sinusoidal spaces were big and most of the cells were darkly stained.

**Figure 14:**
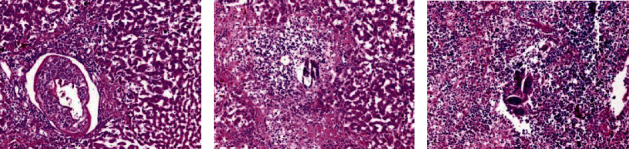
Histological section staining with H&E of infected treated with 600 mg/kg *Origanum majorana* group. (a) Liver showing worm inside portal vein radical with moderate inflammatory changes within the hepatic lobule. (b) Liver showing intralobular ova enclosed by dense inflammatory cellular reaction and focal necrosis. (c) Some ova surrounded by inflammatory cellular reaction in the spleen (×200).

**Figure 15:**
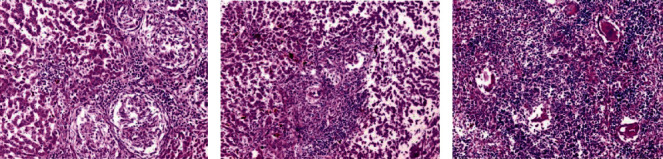
Histological section staining with H&E of infected treated with 600 mg/kg *Ziziphus spina-christi* group. (a) Many lympho-epithelioid granulomas in the liver. (b) Amalgamated ova granulomas in the liver. (c) Many fresh deposited and degenerated egg in the spleen (×200).

**Figure 16:**
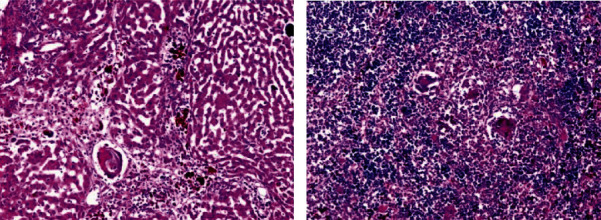
Histological section staining with H&E of infected treated with 800 mg/kg *Salvia fruticosa* group. (a) Degenerated ova with mild infiltration of the hepatic lobule by mononuclear inflammatory cells in the liver. (b) Some degenerated ova, enclosed by lympho-epithelioid cellular inflammatory cellular infiltration (×200).

Histological liver sections treated with *Origanum majorana*, *Ziziphus spina-christi*, and *Salvia fruticosa* appeared temperate diffuse infiltration of liver parenchyma by chronic inflammatory cells without watched eggs or zone fibrosis (Figures 14–16). They appeared nonattendance of fibrosis and bilharzial eggs with an important decrease of infiltration in liver parenchyma by the chronic inflammatory cells. Whereas, spleen sections display more or less devolution of egg, enclosed by infiltration of lympho-epithelioid cellular inflammatory cells.

## 4. Discussion

With the expanding publicity of medications based on aqueous or alcoholic extracts or any other organic dissolvent of natural therapeutic plant extracts, the current study is planned to assess the antischistosomal influence of *Origanum majorana*, *Ziziphus spina-christi*, and *Salvia fruticosa* aqueous extract in the golden hamster experimentally infected with *S. haematobium* in comparison with the present utilized PZQ in patent and prepatent stages of the disease.

The aqueous extracts of *Origanum majorana*, *Ziziphus spina-christi*, and *Salvia fruticosa* were chosen to avoid the high toxicity of organic dissolvents such as chloroform, methanol, dichloromethane, and acetone because water is a safe, nontoxic widespread dissolvent to living cells [34].

In the current study, different concentrations of the impact of *Origanum majorana*, *Ziziphus spina-christi*, and *Salvia fruticosa* aqueous extracts against Egyptian strain of *Schistosoma* (*S. haematobium*) were assessed *in vitro* and *in vivo* in the experimentally infected hamster. The study was the primary one that examined the effectiveness of *Origanum majorana*, *Ziziphus spina-christi*, and *Salvia fruticosa* against *S. haematobium*. Additionally, as a step to begin with, *in vitro* studies of antischistosome carry out on the adult stage and schistosomule.

The current *in vitro* study has been confirmed the antischistosomal efficacy of *Origanum majorana*, *Ziziphus spina-christi*, and *Salvia fruticosa* extract on *S. haematobium* adult stage (survival time, motility, mating, and modifications of tegument) at different concentrations of 500, 250, 125, 62.5, and 31.25 *μ*g/ml.

The effects of dose-dependent were watched, the most efficient one with a concentration of 500 *μ*g/ml begin in a shorter incubation period. All extracts that were examined caused the unpairing of couple worms, moderate constriction, decrease in motility, and loss of motion leading to the parasite's death most of the time, in a study accomplished by Noel [35], clarified the disability-related with significant neuromodulators or neurotransmitters such as acetylcholine, dopamine, and/or serotonin [35].

*Origanum majorana* and *Ziziphus spina-christi* extract were more effective mating inhibitors than *Salvia fruticosa* extracts. *In vitro*, *Origanum majorana* and *Ziziphus spina-christi* extract of (500 mg/kg) were efficient as PZQ. Previously, studies by Pica-Mattoccia and Cioli [36] had stated the PZQ impact on the adult worms, caused permanent contractions whenever the adult worms were uncovered to concentrations of 0.1 and 1 *μ*g/ml. Though, PZQ was known to cause a speedy calcium flow taken after by contraction, loss of motion, and tegument demolition.

The optical inverted microscopy utilizes did not permit the description of tegumental changes displayed in the parasite; in the present study, a specific examination with SEM (scanning electron microscopy) was used to assess the sample tegumental harm after treatment *in vitro*. Many authors had been used SEM in arrange to explain the mechanisms of drug/compound activity utilized in the treatment of schistosomiasis test [37, 38].

The changes caused by aqueous extracts treatment of *Origanum majorana*, *Ziziphus spina-christi*, and *Salvia fruticosa* regard to harm in suckers, oral and acetabularin of both female and male schistosomes. Adult worms investigation with SEM showed that the treatment caused an integument broad peeling, particularly in the dorsal area, occurring from the exposure of these surface antigens. Besides, blebs were visual on the male worms treatment exposed to *Origanum majorana*, *Ziziphus spina-christi*, and *Salvia fruticosa* extracts. Similar results were reported by de Oliveira [39], as the *Schistosoma mansoni* worms that were treated with an aqueous fracture extract and also dichloromethane crude of *Baccharis trimera*, showed strong efficacy in killing adult worms. The sarcoplasmic membrane of schistosomiasis is the first defense wall. Changes in the body wall of adult worms may result after treatment with medicinal plant extracts with antischistosomiasis activity through their membranes, allowing the sarcoplasmic membrane of the adult worms to be exposed to antigens. Younes [40] prepared an ethanolic extract of the edible pomegranate plant, which showed efficacy on the adult worms of *Schistosoma mansoni*. Kang [41] demonstrated that the mice were dosed with hederacolchiside A1 fractional extract from *Pulsatilla chinensis* in their studies on *Schistosoma mansoni* and *Schistosoma japonicum*, where the results showed that the worms were killed after 11 days in mice, and the pathological parameters in mice infected with *Schistosoma japonicum* began to improve.

In the current study, The *Origanum majorana*, *Ziziphus spina-christi*, and *Salvia fruticosa* extracts *in vivo* antischistosomal effect on *S. haematobium* infected golden hamster were assessed concerning histopathological changes (schistosomal granulomas, hepatic inflammation effective, and a number of egg in the tissues of the liver), spleen and kidney. The examined extracts appeared dose-dependent decrease in both granuloma number and diameter, egg number in spleen and liver tissues, both, fibrosis and inflammatory infiltration compared to infected control (the untreated control groups).

The efficiency of *Origanum majorana* and *Ziziphus spina-christi* extracts was more than *Salvia fruticosa* in decreasing granuloma diameter and number. An important decrease appeared in hepatic fibrosis and inflammatory liver infiltration by using *Origanum majorana* extract comparative PZQ. *Ziziphus spina-christi* appeared temperate activity, whereas *Salvia fruticosa* extract appeared less conspicuous effect. *Origanum majorana* extract at the higher treatment appeared significant result compared to PZQ in decreasing diameter and granuloma number. The decrease of granulomatous inflammation volume indicated an anti-inflammatory influence of the utilized extracts. Concerning the eggs of *Schistosoma* in infected hamster tissues, changing reaction was with different extracts, where *Salvia fruticosa* extract decreased the egg number in the spleen and liver tissues at the high concentration, while eggs were found damage or not found in liver and spleen tissues with *Origanum majorana* at the same dosage and *Ziziphus spina-christi* extract at the lower dosage compared to PZQ. The results acquired from the past experiments *in vivo* and *in vitro* performed by the other authors were similar, utilizing *Piper tuberculatum* separated component, 8-hydroxyquinoline derivatives from *Baccharis trimera*, *Artemisia annua*, and pomegranate that have shown effectiveness against schistosome [42–46].

## Figures and Tables

**Table 1:** Identified compounds in aqueous extract of *Origanum majorana.*

No.	RT	M. wt	M. formula	Prediction	Area	Area %
1	20.52	204	C_15_H_24_	Zingiberene	54727666.55	0.36
2	20.80	222	C_15_H_26_O	Nerolidol	79653206.07	0.52
3	26.89	222	C_15_H_26_O	Cubenol	90321047.45	0.59
4	26.89	222	C_15_H_26_O	Eudesmenol	118361109.56	0.78
5	28.42	220	C_15_H_24_O	Cedren	176248647.53	1.16
6	30.66	30.66	C_15_H_22_O	Phenol	98240348.64	0.65
7	30.81	30.81	C_11_H_14_O_3_	Butanone	163365217.48	1.08
8	33.26	222	C_15_H_26_O	Eudesmol	106173887.45	0.70
9	36.84	188	C_9_H_16_O_4_	Propanol	164765320.50	1.08
10	45.71	276	C_17_H_24_O_3_	Gingerol	6205744296.17	40.86
11	47.93	200	C_6_H_9_FN_6O_	Capsaicin	274275241.47	1.81
12	48.83	292	C_21_H_4_0	Nonivamide	151523642.98	1.00
13	60.25	412	C_29_H_48_O	Stigmasterol	92898328.92	0.61
14	61.17	414	C_29_H_50_O	á-Sitosterol	351889991.52	2.32

**Table 2:** Identified compounds in aqueous extract of *Ziziphus spina-christi* (L.).

No.	RT	MW	M. formula	Identified compounds	Chromatogram area	Chromatogram area %
1	13.23	615	C23H45N5O14	D-Streptamine	8421115.74	0.48
2		154,	C10H18O	p-Menthadiene		
3	17.73	592	C16H48O8Si8	Cyclooctasiloxane	36726047.85	2.11
4	19.82	110	C6H6O2	Hydrochinon	54912618.86	3.15
5	20.47	145	C8H7N3	Benzene	70108424.28	4.02
6	20.89	458	C28H30N2O4	Morphinan	28332599.99	1.62
7	24.58	220	C15H24O	Tricycloundecan	16114395.18	0.92
8	24.58	220	C15H24O,	Spathulenol	16114395.18	0.92
9	24.76	153	C6H7N3O2	Imidazole	19324582.43	1.11
10	26.32	190	C13H18O	Megastigmatrienone	17929010.41	1.01
11	26.93	283	C10H13N5O5	Guanosine	22821026.68	1.31
12	32.76	279	C10H17NO6S	Desulphosinigrin	20218112.12	1.16
13	41.28	519	C30H53NO4Si	Glycine	28017325.31	1.61
14	46.99	286	C20H30O	Phenanthrenemethanol	33473432.07	1.92
15	53.07	439	C31H21NS	Thienopyridine	14058118.79	0.81
16	61.17	414	C29H50O	á-Sitosterol	98265259.33	5.63

**Table 3:** Identified compounds in aqueous extract of *Salvia fruticosa.*

No.	RT	MW	M. formula	Identified compounds	Chromatogram area	Chromatogram area %
1	10.43	341	C20H23NO4	Quinolinol	3053550.06	3.26
2	14.16	576	C18H52O7Si7	Tetrasiloxane	3831389.13	4.08
3	20.88	428	C27H44O2Si	Androstadienol	2861964.11	3.05
4	22.53	283	C10H13N5O5	Purinol	1584222.84	1.69
5	30.94	428	C27H40O4	Spirosten	1522237.77	1.62
6	32.36	256	C16H32O2	Tetradecanoic acid	12521180.76	13.35
7	35.11	195	C11H17NO2	Benzenemethanol	4860449.32	5.18
8	36.42	366	C13H30N2O4SSi2	Cystathionine	1306854.22	1.39
9	49.30	514	C27H31BrO5	Terphenyl	2374908.89	2.53

## Data Availability

The data used to support the results of this study were deposited in a repository [Twumasi et al., 2020 doi:10.1371/journal.pntd.0008919; Alves et al., doi:10.1017/S003118202000181X; Yones et al., 2016 10.1155/2016/2872708].
